# A holistic approach to implementing artificial intelligence in radiology

**DOI:** 10.1186/s13244-023-01586-4

**Published:** 2024-01-25

**Authors:** Bomi Kim, Stephan Romeijn, Mark van Buchem, Mohammad Hosein Rezazade Mehrizi, Willem Grootjans

**Affiliations:** 1 House of Innovation (Department of Entrepreneurship, Innovation and Technology), Stockholm School of Economics, Stockholm, Sweden; 2 Radiology, Leiden University Medical Center, Leiden, Netherlands; 3 KIN Center for Digital Innovation, Vrije Universiteit Amsterdam, Amsterdam, Netherlands

**Keywords:** Artificial intelligence, Implementation science, Change management, Information systems, Digital technology

## Abstract

**Objective:**

Despite the widespread recognition of the importance of artificial intelligence (AI) in healthcare, its implementation is often limited. This article aims to address this implementation gap by presenting insights from an in-depth case study of an organisation that approached AI implementation with a holistic approach.

**Materials and methods:**

We conducted a longitudinal, qualitative case study of the implementation of AI in radiology at a large academic medical centre in the Netherlands for three years. Collected data consists of 43 days of work observations, 30 meeting observations, 18 interviews and 41 relevant documents. Abductive reasoning was used for systematic data analysis, which revealed three change initiative themes responding to specific AI implementation challenges.

**Results:**

This study identifies challenges of implementing AI in radiology at different levels and proposes a holistic approach to tackle those challenges. At the technology level, there is the issue of multiple narrow AI applications with no standard use interface; at the workflow level, AI results allow limited interaction with radiologists; at the people and organisational level, there are divergent expectations and limited experience with AI. The case of Southern illustrates that organisations can reap more benefits from AI implementation by investing in long-term initiatives that holistically align both social and technological aspects of clinical practice.

**Conclusion:**

This study highlights the importance of a holistic approach to AI implementation that addresses challenges spanning technology, workflow, and organisational levels. Aligning change initiatives between these different levels has proven to be important to facilitate wide-scale implementation of AI in clinical practice.

**Critical relevance statement:**

Adoption of artificial intelligence is crucial for future-ready radiological care. This case study highlights the importance of a holistic approach that addresses technological, workflow, and organisational aspects, offering practical insights and solutions to facilitate successful AI adoption in clinical practice.

**Key points:**

1. Practical and actionable insights into successful AI implementation in radiology are lacking.

2. Aligning technology, workflow, organisational aspects is crucial for a successful AI implementation

3. Holistic approach aids organisations to create sustainable value through AI implementation.

**Graphical Abstract:**

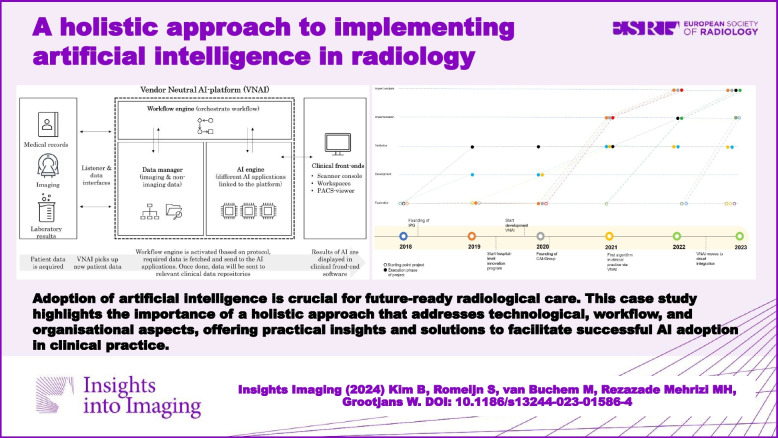

## Objectives

Recent years have seen an upsurge of interest in artificial Intelligence (AI), especially deep learning (DL), for radiology [1]. More than half of CE-marked medical AI devices between 2015 and 2020 were targeted for use in radiology [2], and the number of participating AI companies nearly doubled in RSNA 2019 compared to the year before [3]. Nevertheless, the adoption of AI applications in clinical practice remains limited [4–8].

Studies have shown that the implementation of AI in healthcare, particularly in radiology, faces several challenges spanning technological, workflow and organisational levels. These challenges include the complexity of IT infrastructure [6], the lack of automated data routing and processing [5], limited infrastructure to deploy and scale narrow AI applications [8] and the poor definition of clinical use cases [4]. Additionally, different stakeholders including clinicians and managers have limited knowledge on how to optimally deploy AI [9, 10].

Despite the widespread recognition that overcoming these challenges is key to implementing AI in clinical practice, few efforts have been made to capture how organisations navigate these challenges in practice, especially through a holistic approach. The purpose of this article is to discuss lessons learned from an in-depth case study of one such pioneering organisation and provide applicable insights for organisations seeking to implement AI. In this paper, our focus lies primarily on the application of AI in radiology; however, it is important to note that the holistic approach and its associated lessons are relevant beyond this specific field. We show how an organisation navigated concrete challenges around AI implementation preemptively and with what results. We conclude by providing an overview of the lessons learned for other organisations towards a holistic AI implementation.

## Methods

We performed a longitudinal, qualitative case study to investigate the implementation and deployment of AI applications in radiology in a real-life context [11]. Based on detailed empirical observations, we aimed to develop transferable insights for informing actions in comparable organisational contexts [12, 13]. We selected our research site to be Southern (pseudonym), a large academic medical centre in the Netherlands, because it provided a rich case where the phenomenon of interest is easily observable [14]. Given the scale and scope of its AI-related initiatives (more than 15 ongoing AI implementation projects), Southern qualified as a pioneering case for AI implementation in healthcare within the European context.

Between November 2019 and September 2022, the first author conducted work observations over 43 non-consecutive days, 30 meeting observations, 18 interviews and collected 41 documents that were analysed for this study (see Table 1). To ensure validity in our data, we triangulated both data collection methods and data sources [15].

**Table 1:** Overview of collected data

Data source	Contribution to research
Observations	
• On-site and virtual work observations over 43 non-consecutive days, avg. 4.5 h (e.g. radiology reporting and protocol development in IPG)	• Comprehensively capturing organisational changes related to AI implementation
• 30 meetings, avg. 38 min (e.g. clinical evaluation and threshold optimisation of AI applications)	• Observing the natural progression of AI implementation efforts
Interviews	• Understanding all of technical, clinical, and managerial perspectives and stakes in AI implementation
• 18 interviews, avg. 53 min with clinical, technical, and managerial staff (e.g. AI-related educational efforts, management motivation for AI implementation)	
Documents	
• 19 public documents (e.g. CAI Group blog posts, AI product brochures) and 22 internal documents (e.g. algorithm validation results, planning documents on AI implementation)	

A total of 228 h of data collection (43 days of work observations, 30 meeting observations, 18 interviews) and 41 relevant documents

We systematically analysed our data in three steps following abductive reasoning [16], as described in Table 2. First, we coded concrete change initiatives related to AI implementation at Southern. Next, we coded unique challenges around AI implementation, which were observed at Southern as well as recognised in the literature on AI in radiology. Then, we related the two and removed change initiatives that did not respond to specific AI implementation challenges. The analysis resulted in three themes that span technology, workflow and organisational levels and structure the change initiatives, which are presented in Table 3.

**Table 2:** Data analysis steps

Step	Action	Result
1. Coding concrete change initiatives related to AI implementation at Southern	We analysed each change initiative by asking (a) what was the existing situation and what was not effective about it for (future) AI implementation? (b) what social and technological changes were made? and (c) how did these changes expand the organisation’s capability to implement and use AI more effectively?	A table in Microsoft Excel
2. Coding unique challenges around AI implementation observed at Southern	We analysed each challenge by linking it to ongoing conversations in the literature and identifying whether it relates to the level of technology, workflow or organisation	Table 3 (first column); unique AI implementation challenges spanning technology, workflow and organisational levels
3. Relating concrete change initiatives to AI implementation challenges	We analysed how each change initiative responds to AI implementation challenges across three levels. This helped us identify and exclude change initiatives that are generic in all innovation processes such as having a change agent	Table 3 (second column); three themes that encompass the change initiatives

**Table 3:** Overview of challenges around AI implementation, change initiatives at Southern and learnings based on performed case study

Challenges around AI implementation, observed at Southern and recognised in the literature on AI in radiology	Change initiatives observed at Southern	Lessons learned on a holistic approach to AI implementation from the case of Southern
**Technology level** • Current AI applications have narrow functions, requiring the combined use of multiple applications [8] • Organisations need to work with multiple vendors, each providing a narrow AI application, resulting in considerable overhead costs [17] • There is a lack of standard user interfaces to integrate AI results into the clinical workflow [4]	**Scalable and seamless AI implementation** • VNAI: automatically routes data, reads the metadata and triggers relevant AI applications to orchestrate the use of multiple applications • VNAI: centralises legal and contractual procedures around AI implementation to reduce overhead costs • Development of integrative frameworks to ensure integration into routine clinical software: enables seamless integration of AI results into the clinical workflow	• Initiate change efforts that comprehensively address not only technology but also structure, tasks and personnel involved in integrating AI into clinical practice • Consider integrative platforms such as vendor-neutral AI platforms for integrating and orchestrating multiple AI initiatives • Reduce the cost and time of implementation by defining a holistic framework for the testing, validation, integration, monitoring of AI tools
**Workflow level** • Due to a lack of best practice, the use of how AI applications is highly varied in practice, making it hard to prove their value [18] • AI applications are fitted to specific data, which may not correspond to the patient population of the organisation implementing them [13] • Current AI applications generate results with limited ability to interact with radiologists and receive their feedback real-time [7]	**Value-centric AI implementation** • IPG (radiographers): ensures a standardised and consistent use of technology and centralises expertise on technology use • IPG (technical physicians): leads the local validation, fine-tuning, and continued monitoring of AI applications • Making AI results modifiable: ensure that the end user remains in control and can accept/reject or adjust AI results before they are entered into the PACS system	• Select and prioritise AI implementation projects with a focus on creating value and addressing specific clinical problems. To this end, leverage the expertise and insights of clinicians to identify areas where AI can have the most significant contribution to clinical practice • Ensure seamless integration of AI applications into clinical workflows to facilitate real-world adoption and maximise their impact
**People and organisation level** • Radiologists have diverging expectations of the benefits and risks of AI [19] • Radiologists do not yet have a basic proficiency in AI and machine learning [10] • There is a gap between clinical and technical expertise, creating a barrier to the effective integration of AI technologies in healthcare	**Organisation learning from AI implementation:** • CAI Group: helps radiologists and other clinicians by assessing the viability and necessity of proposed AI projects and providing a comprehensive checklist • CAI Group: fosters learning among data scientists, radiologists and other clinicians as an umbrella organisation, encourages cross-departmental communication and collaboration and disseminates knowledge on AI • Innovation steering committee: streamlines AI projects by centralising and evaluating bottom-up ideas on AI use cases	• Provide a clear organisational structure with dedicated people/roles for AI implementation (as opposed to being a part of the work of clinicians) • Establish dedicated occasions for interaction between technical expertise and clinical knowledge to facilitate effective communication and collaboration between these two critical areas for AI implementation • Make sure to manage local initiatives whilst also considering changes at a more global level and consider reorganising the current workflow to generate more value from AI applications • Encourage cross-departmental (radiology vs other medical departments) knowledge sharing to foster a culture of learning and collaboration around AI implementation

We provide an overview of the AI applications implemented in the radiology department at Southern in Table 4. The table provides a detailed breakdown of each application, including an evaluation of its impact on clinical practice. It shows the wide range and variety of AI applications in Southern, their adoption stage and observed (or potential) impact on clinical practice.

**Table 4:** Overview of integrated AI applications in Southern

Type of AI application	State of adoption/integration	Impact on clinical practice
3D tumour segmentation of vestibular schwannoma on MRI	Ready for prospective validation in research setting	Transition from 2D measurements to automated 3D volume measurement, resulting in time reduction and quality improvement
Normal/abnormal detection chest X-ray	Fully integrated into clinical workflow (validated on prospective data). AI output directly present in worklist of radiologist. Detailed overlay images presented in separated viewer	± 45% of all chest X-ray cases in clinical practice are normal. The algorithm is able to automate reporting for approximately 20% of all normal cases, enhancing clinical efficiency
Lung nodule detection on CT-Thorax	Fully integrated into clinical workflow (validated on prospective data). Output (number of detected lung nodules and percentage of affected lung tissue) presented in PACS worklist and possibility to accept/reject/modify nodule segmentations in PACS viewer	Substantial time reduction in follow-up imaging and improved lung nodule comparisons over time
Bone age measurements on X-ray	Fully integrated into clinical workflow (validated on prospective data). AI report automatically available in PACS	Automated bone age measurements on x-ray, facilitating task differentiation to advanced practitioners
Covid detection and quantification on CT	Fully integrated into clinical workflow (validated on prospective data). AI report automatically available in PACS	Robust quantification of COVID-affected lung parenchyma in all lung segments, significantly enhancing reporting efficiency and quality
Leg angle and distance measurements on X-ray	Fully integrated into clinical workflow (validated on prospective data). AI report is automatically available in PACS. Standardised radiological report based on AI output	Automated leg angle and distance measurements with an AI acceptance rate of approximately 90%
MRI neuro quantification for dementia patients	Fully integrated into clinical workflow (validated on prospective data). AI report automatically available in PACS	Automated quantification of white matter abnormalities and atrophy evaluation
Automatic quality feedback for chest X-ray	Fully integrated in clinical workflow. Automatic quality feedback on iPad after image acquisition	Enhancement of image quality to ensure accurate reporting and prevent the need for patients to return, as low-quality images may otherwise necessitate their return for re-imaging
Large vessel occlusion detection for early stroke detection	Fully integrated into clinical workflow (validated on prospective data)	
Fracture detection on X-ray	Implementation phase. Connected to clinical systems and ready for clinical use	*Potential impact*: decreased reporting time, enhanced diagnostic confidence and subsequently boost job satisfaction, particularly during night and weekend shifts when residents work independently
Scoliosis measurements on X-ray	Implementation phase. Connected to clinical systems and ready for clinical use	Potential impact: automated scoliosis measurements
Automated vertebral fracture assessment on DXA	Development phase, model development and retrospective validation	Potential impact: automated vertebral fracture assessments resulting in significant reduction in reporting time. Prototype has shown positive impact on reader discomfort for annotation
Prostate analysis on MRI	Exploration phase	Potential impact: decreased reporting time by pre-filled structured report based on AI output
AI for tomosynthesis	Exploration phase	Potential impact: decreased reporting time by pre-filled structured report based on AI output
Knee osteoarthritis measurements on X-ray	Exploration phase	Potential impact: automated osteoarthritis measurements. Reduction in reporting time

## Results

In this section, we present our results following the structure of Table 3. We review the unique challenges that Southern faced in its attempt to implement AI and how Southern responded to these challenges with concrete change initiatives.

### Technology level: scalable and seamless AI implementation

**Fig. 1:**
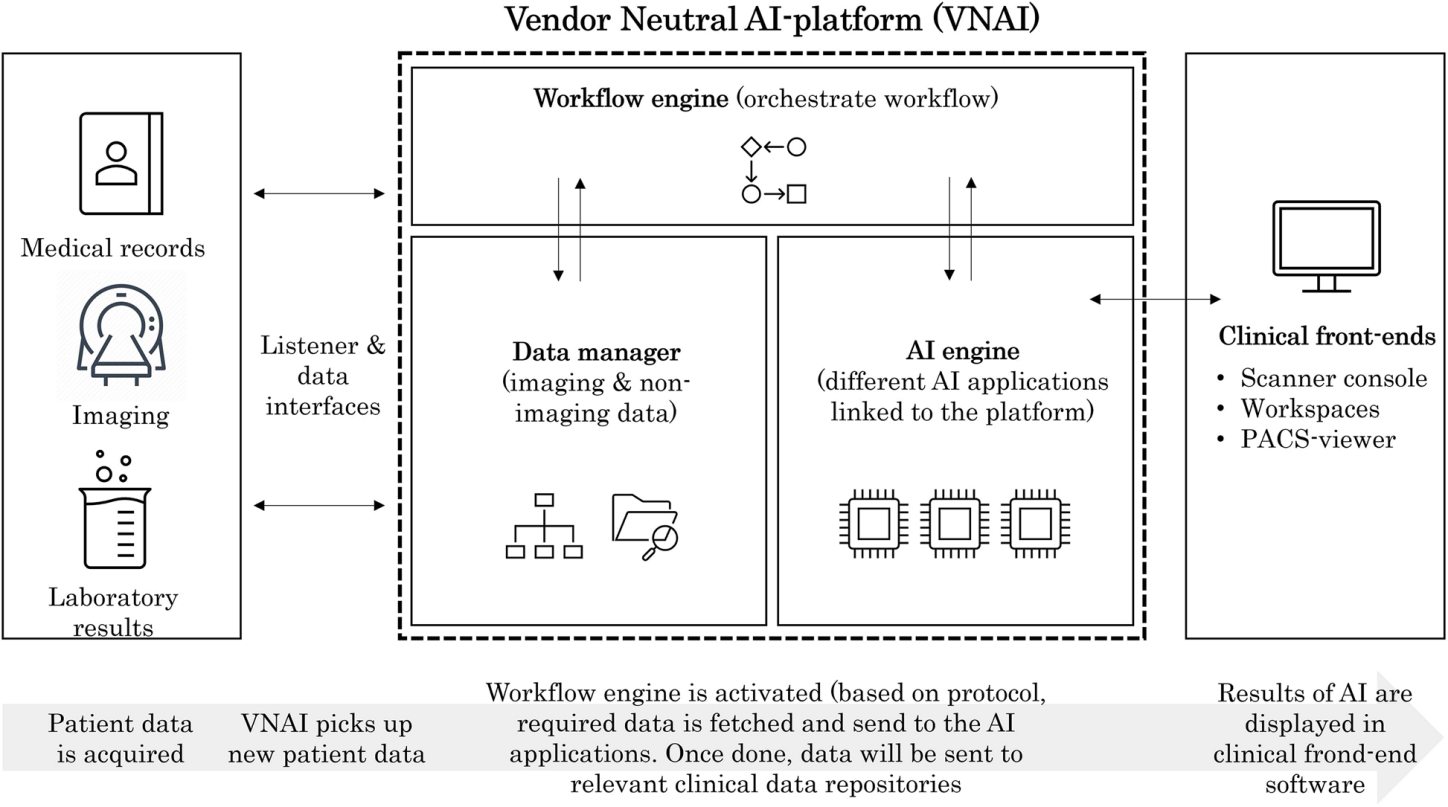
Hospital-level vendor-neutral AI (VNAI) platform towards a scalable and seamless AI implementation

Because current AI applications have narrow functionality, capable of performing specific functions on specific types of data [7], organisations need to orchestrate the combined use of multiple AI applications [8]. Considering this challenge, Southern implemented a hospital-level vendor-neutral AI (VNAI) platform, which enables the upscaling and streamlining of AI implementations and clinical use. For radiology AI applications, the VNAI uses metadata (e.g. imaging modality, scanning protocol, patient age cut-off) of acquired scans and automatically routes eligible data to relevant AI applications for processing as presented in Fig. 1. This way, it can orchestrate the simultaneous use of multiple AI algorithms in the desired sequence, thereby ensuring a workflow-centric AI implementation without the radiologist experiencing workflow disruptions: “I know that all too often, data generated by AI may not be available on the work floor at the right time. That is essential before they can add value.” (Radiologist L).

Another challenge in the current AI landscape is that organisations need to work with multiple vendors, each providing a narrow AI application, which results in considerable overhead costs [17]. The VNAI helps here too by centralising security review, contractual issues, and technical installation of multiple AI projects. It acts as a centralised data processor for all AI applications hosted on the platform, reducing the need for privacy and data security paperwork with individual AI vendors. In this manner, an overarching and fortified regulatory framework is established for all AI projects, consequently yielding faster and less costly AI implementation. To illustrate, the VNAI expedited implementing AI applications to a couple of months, whereas before the VNAI, implementing stand-alone AI applications with individual AI vendors took over a year, especially due to the lengthy process of drafting and signing legal and contractual documents. Additionally, the VNAI only hosts certified AI applications, allowing Southern to quickly instal and test credible AI applications that can be used in clinical practice. In addition, Southern recently moved from an on-premise VNAI to a cloud-based VNAI to facilitate future software upgrades and to shift maintenance work to the VNAI provider, thereby further reducing overhead costs.

Lastly, the lack of standard user interfaces to integrate AI results into clinical front-end software further challenges the implementation and deployment of AI in clinical practice. Suboptimal AI integration diminishes the added value of AI and hinders easy utilisation by end-users [4, 17, 18]. To tackle this challenge, Southern prioritised seamless integration of AI applications into their clinical front-end software (e.g. PACS viewer), allowing clinicians to seamlessly access and view AI results in their viewer with minimum additional clicks. This reduces workflow disruptions, freeing clinicians from having to fire up separate software and switch between interfaces. Whilst a superficial URL integration, which fires up a separate web browser with AI results, would result in a quicker integration of any individual AI application, managers and clinicians at Southern were adamant that it was not scalable nor sustainable. Thus, they pushed for seamless integration of AI into clinical front-end software as a prerequisite for their clinical use. This was done through co-creation with AI vendors to get specific AI outputs and through configuration of APIs of existing clinical front-end software applications (e.g. PACS and other advanced image viewers).

**Fig. 2:**
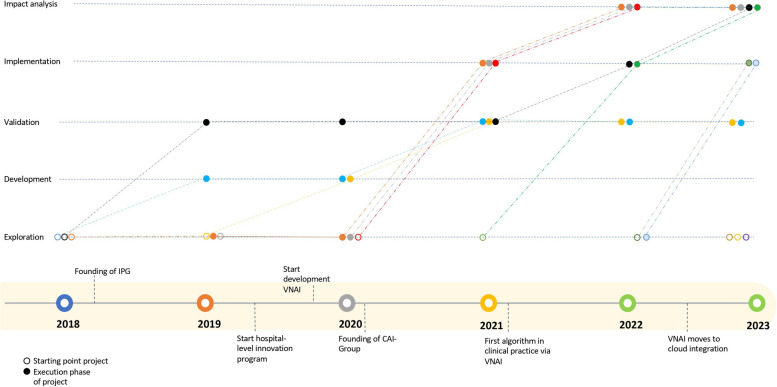
Overview of progress of AI projects over time. Open dots represent the starting point of the project, whereas solid dots represent the progress of the projects over time. Important events are presented on the timeline at the bottom of the figure

Figure 2 summarises the evolution of AI projects within the radiology department of Southern. Southern started exploring various AI applications in 2018. Several projects stayed for a whilst in the development and validation phase in 2019 and 2020. However, after the VNAI became available in 2020, multiple projects accelerated and resulted in the first implementation of AI in clinical practice. The acceleration, although for the significant part, was not entirely due to the VNAI, and it should be noted that multiple internal and external factors were at play, such as availability of resources from AI vendors, IT infrastructure and knowledge on legal and ethical issues.

### Workflow level: value-centric AI implementation

Because there is no guideline regarding how best to implement AI into clinical workflows, their use in clinical practice shows significant variations [4, 17, 18]. As a result, organisations struggle to establish empirical evidence on the value of AI applications. Managers at Southern knew that to tackle this challenge, AI applications needed to be used in a standardised, consistent manner: “Although costing a fortune, image analysis tools were not being used in a consistent manner.” (chairman, department of radiology). To this end, Southern established the Image Processing Group (IPG, pseudonym) inside the radiology department in 2018 as a central hub for technology deployment and integration. In the IPG, nine radiographers and two technical physicians deploy and integrate advanced technologies including, but not limited to, AI. Using technology, radiographers analyse radiological images and pre-populate radiological reports before radiologists report on the case. In doing so, they apply technology in a structured and standardised manner through the use of detailed protocols. The IPG further realises value from technology by not merely using technology but reorganising work around it and shifting tasks from radiologists to radiographers. Lastly, the IPG also fosters technology expertise by being a central hub for technology use: “We have more knowledge on [image analysis software] to make quick changes. What would take me just a couple of minutes, the radiologist needs to turn on the program, figure out where the tool is, how to use it, etc.” (IPG radiographer).

Meanwhile, unlike other software, AI applications are trained on specific datasets. Hence, their performance can vary when the training data and the local data it is being applied to are different (e.g. patient population, prevalence of pathologies) [20]. To tackle this challenge, the two technical physicians in the IPG play an important role of locally validating the algorithms, measuring their performance metrics such as accuracy, precision and continuously evaluating their performance over time. Furthermore, they communicate the wishes of clinicians to AI vendors and are responsible for the seamless integration of AI in the clinical workflow. For instance, they collaborated with the AI vendor to perform a retrospective analysis on over 15,000 chest X-rays from Southern to validate an AI application providing normal/abnormal detection on chest X-rays, which was developed in a different country. After the successful retrospective analysis of the algorithm together with the vendor, they created a dashboard to automatically analyse findings from radiology reports and compare them to the AI results. The dashboard enables managers to monitor AI results and enables radiologists to control the quality of their work. Throughout this process, the IPG technical physicians’ multidisciplinary background in medicine and engineering facilitated the coordination and collaboration of stakeholders within and beyond Southern.

Finally, current AI applications have limited ability to interact with radiologists and receive their feedback real-time [7]. To tackle this challenge, radiologists at Southern demanded modifiable AI results so that they can have more control over radiological reports. Making AI results modifiable usually requires the interface of multiple software packages to communicate, such as the PACS viewer and the AI software. Since software interfaces usually have different communication standards and implementations, managing such communication requires an intensive and prolonged collaboration with the vendors of all software packages. To illustrate, in 2021, Southern implemented an AI application which detects and quantifies lung nodules on CT scans in 2021. Initially, the AI results were presented in a PDF file in PACS, with findings that were fixed. To make AI results modifiable, Southern collaborated with both the AI and PACS vendors and used existing application programming interfaces (APIs) to enable an accept/reject module to be used as well as to adjust measurement in the PACS viewer. Although this delayed the integration by several months, radiologists were more convinced of the value that the AI application adds to their work and, as a result, more eager to use the AI application.

### People and organisation level: continuous learning from AI implementation

Radiologists have diverging expectations of the benefits and risks that AI can bring to their work [19], partly due to having limited experience with AI in clinical practice: “there were a lot of talks, a lot of presentations going on about AI, but in practice, you never get in touch with AI.” (radiology resident). To help radiologists and other clinicians with limited experience select and invest in the right AI projects, Southern established the Clinical AI Implementation Group (CAI Group, pseudonym) in 2020. When those interested in AI projects submit their proposal, data scientists, legal and ethical experts and clinicians in the CAI Group altogether assess the viability of the proposal, if there are enough resources and if there is concrete value added from using AI. More fundamentally, it is assessed whether AI is required to solve the clinical question in the first place or if other technology can answer the question as well. The CAI Group also provides clinicians with a comprehensive and concrete checklist for the entire AI cycle, including guidelines on privacy considerations and handling of ethical issues.

Additionally, the CAI Group fostered learning among radiologists and other clinicians and prevented them from reinventing the wheel. It actively disseminates lessons learned from various AI projects through multiple communication channels such as blog posts, meetups and internal newsletters. Particularly, by being an umbrella organisation and affiliating diverse actors who are members of various departments, it facilitates cross-departmental communication and mobilises a broad web of knowledge and other resources across formal departments more easily: “For a trustworthy implementation of AI in clinical practice, we need to bundle hospital-wide knowledge. CAI Group is a perfect example of this. This way we can achieve our goal of scaling AI within the hospital.” (Board member of Southern).

Lastly, to streamline the implementation of AI projects, Southern established an extended network by nominating AI champions from each subspecialty in the radiology department (e.g. neuroradiology). The AI champions gather ideas on potential AI use cases from their subspecialty through internal discussions, which are centralised in the Innovation Steering Committee for further investigation and prioritisation. When a project is selected, a project team is created consisting of clinicians and IT personnel to implement and evaluate the idea gathered through a bottom-up approach. The aim is not to have one-off AI implementations but to establish a feedback loop which leads to peer-to-peer teaching and informs future decisions on AI. This streamlined procedure has resulted in radiologists bringing more realistic use cases and having grounded expectations regarding AI applications.

## Discussion

In this paper, we present a holistic approach to AI implementation by drawing on the case of Southern, a large academic medical centre in the Netherlands, which successfully implemented multiple AI applications over the course of 5 years. Their success is evidenced by the broad range of AI applications already adopted or in use and their consistent integration as well as expedited process and reduced efforts for testing, validating, implementing and monitoring AI applications (see Fig. 2 and Table 4). In this section, we discuss how the change initiatives observed at Southern illustrate a holistic approach to AI implementation and derive lessons learned that can be helpful for other organisations.

With a holistic approach to implementing AI, organisations comprehensively reconsider, restructure and realign technology, workflows, people and organisational structures to create and sustain value with AI. In doing so, they go beyond the possibilities offered by the status quo, as opposed to simply inserting an AI application to legacy systems that may only temporarily improve the status quo without creating lasting value. AI implementation can be at risk of lapsing back to pre-existing states of performing medical tasks, so it is important to ensure that long-term initiatives are in place to keep the change process moving forward. The case of Southern shows that organisations can reap more benefits by investing in long-term initiatives that holistically align both social and technological aspects of clinical practice. Across all the change initiatives of Southern, we observe the following three general patterns. Firstly, efforts are centralised and stakeholders are gathered to foster an effective selection of AI projects, efficient use of AI applications, cross-departmental knowledge sharing and collaboration and expertise on technology use. Secondly, long-term investments are made in AI implementation infrastructure that standardises and automates processes to prevent recurrent (overhead) costs and reinventing the wheel. Lastly, intended end-users are included from the start so that AI implementation delivers value in everyday clinical practice. In Table 3, we summarise the results of our case study and provide more detailed lessons learned on the holistic approach to AI implementation that can be helpful to other organisations.

### Limitations and future research

This research is not without limitations. Firstly, we delved into a single case to derive transferable insights that are rich in context, on a holistic approach to AI implementation. The insights and change initiatives are not aimed to be directly generalisable for different situations but transferable through concepts and lessons that can be learned about a holistic approach to AI implementation [12, 21]. Hence, although we believe that these insights could facilitate AI implementation in different organisations and help realise sustainable value from AI, readers should note that the insights and change initiatives are tuned to the specific organisational and historical context of the selected case.

Relatedly, we do not argue that the holistic approach to AI implementation is the best nor the only approach for all cases. The holistic approach is suitable for organisations with complex workflows and information systems such as tertiary medical institutions, where locally implementing AI applications can produce unforeseen problems due to misalignments between different elements interacting in the organisation. We acknowledge that a local implementation of AI may suffice, for example in organisations with uniform or parallel workflows with smaller scope of operations where implementing a limited number of AI applications can already generate significant value for the organisation. Future research could study multiple cases to investigate under which conditions local implementation of AI may suffice and where the threshold between the two approaches lies in practice.

Lastly, the financial sustainability of the proposed holistic approach to AI implementation is still a separate topic to be addressed. Whilst increasing the number of local-implementation AI applications lacks financial sustainability and scalability, the question remains as to if and when the higher (initial) investments (in both personnel and technology) required for the holistic approach will break even. Our case shows that a holistic approach, especially when data, workflow and algorithms are integrated through platforms (e.g. VNAI) can reduce the costs of validation and implementation, hence contributing to financial sustainability. We also notice that this might be a potential business model for AI companies to provide their services through these platforms, thereby reducing their costs and becoming more financially affordable for user organisations.

It is important to recognise that quantifying the precise financial impact and benefits is a complex and multifaceted issue in itself and requires dedicated research efforts. It is therefore important to note that this specific issue is beyond the scope of this paper. Hence, in addition to the socio-technical alignment addressed in this paper, future research should look into issues of financial sustainability and scalability for a truly wide-scale AI implementation to be achieved.

## Data Availability

All relevant data can be made available on reasonable request to the corresponding author.

## Abbreviations

AI: Artificial intelligence
CAI-Group: Clinical AI Implementation Group
DL: Deep learning
IPG: Image Processing Group
PACS: Picture Archiving and Communication System
VBR: Value-based radiology
VNAI: Vendor-neutral AI
